# US Adults’ Perceptions About the Harms of Nicotine in Electronic Vapor Products on the Adolescent Brain, United States, 2016–2017

**DOI:** 10.5888/pcd17.190391

**Published:** 2020-03-26

**Authors:** Henraya McGruder, Kimp Walton, Saida Sharapova, Brian A. King

**Affiliations:** 1 Office on Smoking and Health, National Center for Chronic Disease Prevention and Health Promotion, Centers for Disease Control and Prevention, Atlanta, Georgia

## Abstract

We used data from the 2016 and 2017 SummerStyles survey (N = 4,186 and 4,066, respectively) to assess US adults’ perceptions about the harms of nicotine in electronic vapor products (EVP) to the developing adolescent brain. Of respondents in 2016, 68.5% agreed exposure to nicotine in EVP was harmful, and of respondents in 2017, 62.6% agreed (*P* < .001). This agreement varied by several covariates. Continued efforts are warranted to educate the public about the risks of EVP use among youth, including the harmful effects of nicotine exposure on the developing adolescent brain.

**Summary**

**What is already known on this topic?**

Electronic vapor products (EVP), including e-cigarettes, are the most commonly used tobacco products among US middle and high school students, and they typically contain nicotine, which is highly addictive and can harm the developing adolescent brain.

**What is added by this report?**

We assessed adults’ perceptions of the harms of nicotine on the adolescent brain and found that two-thirds of US adults agree that nicotine in EVP is harmful; however, variations in agreement were found across subpopulations.

**What are the implications for public health practice?**

Continued efforts are warranted to educate the public about the harmful effects of nicotine on the developing adolescent brain and about the risk of EVP use among adolescents.

## Objective

Electronic vapor products (EVP), including e-cigarettes, have been the most commonly used tobacco products among US middle and high school students since 2014 (1). EVP typically contain nicotine, which is highly addictive and can harm the developing adolescent brain (2,3). Adults serve a critical role in preventing youth tobacco product use (4). Therefore, it is important to educate adults, particularly those who are influencers of youth, about the harmful effects of youth EVP use. We assessed adults’ perceptions about the harms of nicotine in EVP to the adolescent brain by using data from cross-sectional internet surveys conducted in 2016 and 2017.

## Methods

We used data from 2016 (N = 4,186; response rate, 68%) and 2017 (N = 4,066; response rate, 74%) during June and July of both years from SummerStyles, an internet panel survey among adults aged 18 years or older fielded by Porter Novelli (Omnicon Group, New York, New York). Data were weighted to the US adult population based on sex, age, annual household income, race/ethnicity, household size, education, US region, metropolitan status, and internet access.

Perceptions about the harm of nicotine in EVP on the developing adolescent brain were assessed by the question, “Please indicate how much you agree or disagree with the following statement: Nicotine in electronic vapor products can harm a teenager’s developing brain.” Response options were “strongly disagree,” “somewhat disagree,” “neither agree nor disagree,” “somewhat agree,” and “strongly agree.”

Perceptions were assessed using point estimates and 95% confidence intervals; χ^2^ tests were used to determine significant (*P* < .05) differences. Assessed covariates were sex, age, race/ethnicity, educational attainment, annual household income, marital status, US region, children <18 years of age living in household, cigarette smoking status, and EVP use status.

Current cigarette smokers were defined as respondents who smoked at least 100 cigarettes in their lifetime, and smoked cigarettes “every day” or “some days” when surveyed. Former cigarette smokers were respondents who smoked at least 100 cigarettes in their lifetime, and who smoked “not at all” when surveyed. Never cigarette smokers had not smoked 100 cigarettes in their lifetime.

Current EVP users were defined as respondents who ever used an EVP (“e-cigarettes, e-cigars, e-pipes, vape pens, e-hookahs, and hookah pens, such as blu, NJOY, or Starbuzz”) even once and used EVP within the 30 days preceding the survey. Former EVP users were respondents who ever used an EVP, but not within the past 30 days. Never EVP users were respondents who reported never using an EVP, even just one time.

## Results

In 2017, 62.6% of adults agreed (“strongly agree” = 35.7% and “somewhat agree” = 26.9%) that nicotine in EVP harmed the developing adolescent brain; 3.7% somewhat disagreed, 4.4% strongly disagreed, and 29.3% neither agreed nor disagreed (Table 1). Prevalence of agreement was higher among women (65.2%) than men (59.9%) (*P* < .05), and ranged from 61.1% among 25- to 44-year-olds to 65.0% among adults aged 65 years or older (Table 2). By race/ethnicity, agreement ranged from 55.1% among non-Hispanic blacks to 64.5% among non-Hispanic whites. Prevalence of agreement generally increased with greater educational attainment and annual household income. Prevalence of agreement was higher among married adults (64.2%) than single adults (58.9%). By region, prevalence of agreement ranged from 60.5% in the South to 66.0% in the Midwest. Prevalence of agreement was higher among those who lived in households with children (64.4%) compared with those who did not (62.0%). Prevalence of agreement was 42.6% among current smokers, 60.2% among former smokers, and 68.9% among never smokers. Additionally, prevalence of agreement was 34.9% among current EVP users, 50.4% among former EVP users, and 65.4% among never EVP users.

**Table 1:** Adult Perceptions About Whether Exposure to Nicotine in EVP Is Harmful to the Developing Adolescent Brain, SummerStyles Survey, United States, 2017

Characteristic	No.	Strongly Disagree	Somewhat Disagree	Neither Agree/Disagree	Somewhat Agree	Strongly Agree
		% (95% CI)				
Overall	4,066	4.4 (3.7–5.1)	3.7 (3.1–4.4)	29.3 (27.8–30.9)	26.9 (25.4–28.5)	35.7 (34.1–37.4)
**Sex^a^**						
Male	1,981	3.9 (2.9–5.0)	4.1 (3.2–5.3)	32.2 (29.8–34.5)	27.1 (24.9–29.4)	32.8 (30.4–35.2)
Female	2,085	4.8 (3.9–6.0)	3.2 (2.5–4.2)	26.7 (24.6–28.9)	26.8 (24.7–29.0)	38.4 (36.2–40.7)
**Age, y^a^**						
18–24	259	3.6 (1.9–7.0)	3.8 (2.1–6.8)	28.9 (23.5–35.0)	31.3 (25.7–37.6)	32.4 (26.6–38.7)
25–44	1,335	5.5 (4.3–7.1)	4.7 (3.5–6.2)	28.7 (26.1–31.6)	27.4 (24.9–30.1)	33.6 (30.9–36.4)
45–64	1,707	3.7 (2.8–4.9)	3.5 (2.7–4.6)	30.2 (27.9–32.7)	24.6 (22.4–26.9)	38.0 (35.5–40.5)
≥65	765	3.9 (2.6–5.6)	2.2 (1.3–3.7)	29.0 (25.6–32.6)	27.6 (24.2–31.2)	37.4 (33.8–41.2)
**Race/ethnicity**						
Non-Hispanic white	2,976	4.2 (3.4–5.1)	3.6 (2.9–4.4)	27.8 (26.1–29.6)	27.4 (25.7–29.2)	37.0 (35.2–38.9)
Non-Hispanic black	363	4.6 (2.6–7.9)	3.4 (1.9–6.1)	36.9 (31.6–42.5)	22.7 (18.4–27.6)	32.5 (27.4–38.0)
Hispanic	492	5.6 (3.7–8.3)	3.9 (2.4–6.1)	30.2 (25.9–34.8)	26.7 (22.6–31.4)	33.7 (29.2–38.4)
Non-Hispanic other	235	3.2 (1.5–6.9)	4.7 (2.5–8.6)	28.6 (22.4–35.7)	29.6 (23.3–36.8)	33.9 (27.3–41.2)
**Education^a^**						
<High school	256	6.8 (4.1–11.1)	4.4 (2.4–7.9)	35.9 (29.9–42.4)	24.1 (19.0–30.2)	28.9 (23.2–35.2)
High school diploma	1,276	4.0 (2.9–5.3)	3.3 (2.3–4.6)	33.4 (30.6–36.3)	25.1 (22.6–27.8)	34.4 (31.6–37.3)
Some college	1,217	3.7 (2.7–5.1)	4.0 (3.0–5.5)	30.7 (27.9–33.7)	28.4 (25.7–31.4)	33.1 (30.3–36.0)
College degree or more	1,317	4.4 (3.4–5.8)	3.5 (2.5–4.7)	21.8 (19.4–24.3)	28.4 (25.8–31.1)	42.0 (39.1–44.9)
**Annual household income, $^a^**						
<25,000	406	5.0 (3.1–8.2)	3.8 (2.1–6.5)	38.4 (33.4–43.7)	21.9 (17.8–26.6)	30.9 (26.2–36.1)
25,000–49,999	921	2.8 (1.9–4.2)	4.7 (3.3–6.6)	30.7 (27.4–34.1)	25.6 (22.6–28.9)	36.1 (32.8–39.6)
50,000–99,999	1,343	4.7 (3.5–6.1)	3.3 (2.4–4.6)	29.8 (27.2–32.6)	28.5 (25.9–31.3)	33.7 (31.0–36.5)
≥100,000	1,387	4.7 (3.6–6.1)	3.3 (2.5–4.5)	23.6 (21.3–26.2)	28.7 (26.1–31.4)	39.7 (36.9–42.6)
**Marital status**						
Married/living with partner	2,813	4.1 (3.3–4.9)	3.7 (3.0–4.5)	28.1 (26.3–30.0)	26.7 (24.9–28.5)	37.5 (35.5–39.4)
Single	713	5.0 (3.4–7.2)	4.1 (2.7–6.0)	32.0 (28.4–35.9)	28.1 (24.6–31.9)	30.8 (27.2–34.7)
Divorced/widowed/separated	540	4.7 (3.0–7.3)	2.9 (1.7–4.9)	30.2 (26.1–34.7)	26.0 (22.0–30.4)	36.2 (31.8–40.8)
**US region**						
Northeast	779	4.4 (3.0–6.4)	4.4 (3.1–6.3)	28.9 (25.4–32.6)	26.7 (23.3–30.4)	35.6 (32.0–39.4)
Midwest	887	4.7 (3.2–6.7)	3.1 (2.0–4.8)	26.3 (23.1–29.7)	29.0 (25.8–32.5)	36.9 (33.5–40.5)
South	1,480	4.3 (3.2–5.6)	4.3 (3.2–5.6)	31.0 (28.4–33.7)	25.1 (22.7–27.6)	35.5 (32.8–38.2)
West	920	4.2 (3.0–5.9)	2.7 (1.8–4.1)	29.7 (26.5–33.2)	28.3 (25.1–31.7)	35.1 (31.7–38.6)
**Children <18 y living in household^a^**						
Yes	1,335	5.8 (4.3–7.6)	4.1 (3.0–5.6)	25.7 (23.0–28.6)	27.1 (24.5–29.9)	37.2 (34.4–40.2)
No	2,723	3.7 (3.0–4.6)	3.5 (2.8–4.4)	30.7 (28.8–32.7)	26.9 (25.1–28.8)	35.1 (33.2–37.1)
**Cigarette smoking status^a^^,^ b**						
Never	2,319	4.2 (3.4–5.3)	2.9 (2.3–3.8)	23.9 (22.0–25.9)	28.5 (26.4–30.6)	40.5 (38.2–42.7)
Former	1,090	4.1 (2.9–5.8)	4.8 (3.5–6.6)	31.0 (28.0–34.1)	28.1 (25.2–31.1)	32.1 (29.2–35.2)
Current	523	5.4 (3.5–8.3)	4.5 (2.9–7.0)	47.5 (42.6–52.3)	21.5 (17.9–25.7)	21.1 (17.3–25.4)
**EVP use status^a^^,^ c**						
Never	3,471	4.2 (3.5–5.1)	3.1 (2.5–3.8)	27.3 (25.7–29.0)	26.9 (25.2–28.6)	38.5 (36.7–40.3)
Former	461	4.7 (2.8–7.7)	6.0 (3.8–9.1)	38.9 (34.1–44.0)	29.1 (24.6–34.1)	21.3 (17.4–25.8)
Current	121	6.4 (2.9–13.5)	10.9 (6.1–18.8)	47.8 (38.0–57.9)	21.5 (14.3–30.9)	13.4 (8.0–21.7)

Abbreviations: CI, confidence interval; EVP, electronic vapor products.

a Significant χ^2^ test (*P* < .05) across favorability categories within the specified characteristic.

b Current smoking was defined by having smoked at least 100 cigarettes and currently smoking “some days” or “every day.” Former smoking was defined by having smoked at least 100 cigarettes and currently smoking “not at all.” Never smoking was defined as not having smoked 100 cigarettes.

c EVP use was defined as “Electronic vapor products (eg, e-cigarettes, e-hookahs, e-cigars, e-pipes, hookah pens, vape pens, or some other electronic vapor product).” Ever EVP use was defined as ever trying EVP, even just once; current use was any use in the past 30 days; former use was defined as ever use and no use in the last 30 days; and never use was defined as never trying.

**Table 2:** Adult Perceptions About Whether Exposure to Nicotine in EVP Is Harmful to the Developing Adolescent Brain, SummerStyles Survey, United States, 2016 and 2017

Characteristic	2016		2017		*P* Value^b^
	No.	% Agree (95% CI)^a^	No.	% Agree (95% CI)^a^	
**Overall**	4,186	68.5 (66.7–70.1)	4,066	62.6 (61.0–64.3)	<.001
**Sex**					
Male	1,984	65.5 (62.9–67.9)	1,981	59.9 (57.4–62.3)	.002
Female	2,202	71.2 (68.8–73.5)	2,085	65.2 (62.9–67.5)	<.001
**Age, y**					
18–24	263	71.3 (65.0–76.9)	259	63.7 (57.4–69.5)	.08
25–44	1,212	68.1 (64.9–71.1)	1,335	61.1 (58.1–64.0)	.001
45–64	1,757	67.0 (64.3–69.6)	1,707	62.5 (60.0–65.0)	.02
≥65	954	69.9 (66.3–73.2)	765	65.0 (61.2–68.6)	.06
**Race/ethnicity**					
Non-Hispanic white	3,093	68.5 (66.5–70.4)	2,976	64.5 (62.5–66.3)	.004
Non-Hispanic black	422	63.4 (57.8–68.7)	363	55.1 (49.5–60.7)	.04
Hispanic	465	68.6 (63.3–73.4)	492	60.4 (55.5–65.1)	.02
Non-Hispanic other	206	75.6 (67.7–82.0)	235	63.5 (56.2–70.2)	.02
**Education**					
<High school	276	62.3 (55.7–68.5)	256	53.0 (46.4–59.5)	.047
High school	1,242	62.9 (59.7–66.0)	1,276	59.4 (56.4–62.4)	.12
Some college	1,269	70.7 (67.6–73.6)	1,217	61.5 (58.4–64.6)	<.001
College degree or more	1,399	74.4 (71.5–77.0)	1,317	70.3 (67.6–73.0)	.04
**Annual household income, $**					
<25,000	719	57.9 (53.3–62.4)	406	52.8 (47.4–58.1)	.15
25,000–49,999	1,002	68.1 (64.5–71.5)	921	61.8 (58.2–65.2)	.01
50,000–99,999	1,343	69.9 (66.9–73.8)	1,352	62.2 (59.3–65.0)	<.001
≥100,000	1,122	73.6 (70.4–76.5)	1,387	68.4 (65.6–71.0)	.01
**Marital status**					
Married/living with partner	2,616	71.3 (69.2–73.3)	2,813	64.2 (62.2–66.1)	<.001
Single	801	65.1 (61.2–68.9)	713	58.9 (54.9–62.9)	.03
Divorced/widowed/separated	769	63.5 (59.0–67.7)	540	62.2 (57.5–66.6)	.68
**US region**					
Northeast	777	66.9 (62.8–70.7)	779	62.3 (58.4–66.0)	.10
Midwest	1,023	66.5 (62.9–69.9)	887	66.0 (62.3–69.4)	.83
South	1,494	66.5 (63.6–69.4)	1,480	60.5 (57.7–63.3)	.004
West	892	74.6 (71.0–77.9)	920	63.3 (59.7–66.8)	<.001
**Children <18 y living in household**					
Yes	1,391	72.2 (69.0–75.2)	1,335	64.4 (61.3–67.4)	<.001
No	2,791	66.9 (64.8–68.9)	2,723	62.0 (60.0–64.0)	<.001
**Cigarette smoking status^c^**					
Never	2,401	74.3 (72.1–76.4)	2,319	68.9 (66.8–71.0)	<.001
Former	1,172	65.2 (62.0–68.3)	1,090	60.2 (56.8–63.4)	.03
Current	511	47.2 (42.0–52.4)	523	42.6 (37.9–47.5)	.20
**EVP use status^d^**					
Never	3,609	70.8 (69.0–72.6)	3,471	65.4 (63.6–67.2)	<.001
Former	450	59.4 (53.9–64.7)	461	50.4 (45.3–55.6)	.02
Current	117	31.9 (22.4–43.1)	121	34.9 (26.0–45.0)	.68

Abbreviations: CI, confidence interval; EVP, electronic vapor products.

a Agree was defined as “somewhat agree” and “strongly agree” responses.

b Significant χ^2^ test (*P* < .05).

c Current smoking was defined by having smoked at least 100 cigarettes and currently smoking “some days” or “every day.” Former smoking was defined by having smoked at least 100 cigarettes and currently smoking “not at all.” Never smoking was defined as not having smoked 100 cigarettes.

d EVP use was defined as “Electronic vapor products (eg, e-cigarettes, e-hookahs, e-cigars, e-pipes, hookah pens, vape pens, or some other electronic vapor product).” Ever EVP use was defined as ever trying EVP, even just once; current use was any use in the past 30 days; former use was defined as ever use and no use in the last 30 days; and never use was defined as never trying.

The prevalence of agreement that nicotine harms the adolescent developing brain was higher in 2016 (68.5%) than 2017 (62.6%, *P* < .001) (Table 2). By covariates, prevalence of agreement was higher in 2016 compared with 2017 among males (65.5% to 59.9%; *P* = .002), females (71.2% to 65.2%; *P* < .001), 25- to 44-year-olds (68.1% to 61.1%; *P* = .001), and 45- to 64-year-olds (67.0% to 62.5%; *P* = .02). Differences in the prevalence of agreement also existed by education, annual household income, marital status, US region, children living in the household, cigarette smoking status, and EVP use status between 2016 and 2017.

## Discussion

We found that approximately two-thirds of adults in the United States agree that nicotine in EVP is harmful to the developing adolescent brain. However, variations in agreement exist across subpopulations, with lower prevalence among current and former smokers and e-cigarette users.

In 2016, the Surgeon General concluded that the use of products containing nicotine in any form among youth, including in e-cigarettes, is unsafe (2). At that time, the Surgeon General released a Public Service Announcement warning about these risks (5). Subsequently, several states and communities developed educational and media materials to reflect the growing body of scientific evidence on this issue (6). Such information is important given that current e-cigarette use increased 78% among US high school students during 2017–2018 alone (7); this increase was likely because of the recent popularity of newer e-cigarettes such as JUUL, which can be used discreetly, have a high nicotine content, and come in youth appealing flavors (4).

Prevalence of agreement varied between 2016 and 2017, both overall and across subpopulations. This difference could be due to multiple factors, including differences in exposure to media campaigns and education about the risks of nicotine among youth, or differences in respondent characteristics or sample size between the 2 surveys.

This study has limitations. First, the survey was internet-based and may not be fully representative of the US adult population; however, data were weighted to US Current Population survey proportions. Second, data were self-reported, which could lead to recall bias.

In conclusion, about one-third of adults do not agree that nicotine harms the developing brain, which continues to develop through adolescence and into young adulthood (4). Continued efforts are warranted at the national, state, and local levels to educate the public about the risks of EVP use among youth, specifically related to the risks of nicotine exposure (2–4).
